# Prognostic Value of Perioperative Anemia in Neurosurgical‐Oncological Treatment of Spinal Metastases

**DOI:** 10.1002/ijc.70513

**Published:** 2026-04-27

**Authors:** Marija Janjic, Logman Khalafov, Juliane Dittmer, Saif‐Eldin Abedellatif, Tim Lampmann, Harun Asoglu, Mohammed Jaber, Haitham Alenezi, Muriel Heimann, Matthias Schneider, Motaz Hamed, Marcus Thudium, Hartmut Vatter, Mohammed Banat

**Affiliations:** ^1^ Department of Neurosurgery University Hospital Bonn Bonn Germany; ^2^ Department of Anesthesiology University Hospital Bonn Bonn Germany

**Keywords:** complications, perioperative hemoglobin, spinal metastasis, surgical treatment

## Abstract

Neurosurgical resection of spinal metastases is an established treatment option for selected patients with advanced malignancies. However, high perioperative morbidity and mortality may further compromise outcomes in this vulnerable population, emphasizing the need to identify modifiable prognostic factors. Perioperative anemia is common and clinically relevant, yet its impact in this setting remains insufficiently studied. This retrospective, single‐center study included 279 patients who underwent surgical treatment for spinal metastases between 2013 and 2025. Patients were stratified into three groups based on perioperative hemoglobin levels. Multivariable logistic regression, Cox proportional hazards models, and Kaplan–Meier analyses were used to assess associations between perioperative hemoglobin levels and postoperative complications, local recurrence, recurrence‐free survival, and mortality at 1 month and 1 year after surgery. Preoperative anemia was independently associated with an increased risk of hospital‐acquired complications (odds ratio [OR] 2.31, 95% confidence interval [CI] 1.35–3.97, *p* = 0.002), while higher hemoglobin levels showed a protective effect. No significant association was found between perioperative hemoglobin levels and surgical complications (OR 0.73, 95% CI 0.36–1.51, *p* = 0.400). Postoperative hemoglobin levels and perioperative hemoglobin changes were not significantly associated with complications (OR 1.008, 95% CI 0.990–1.026, *p* = 0.389) or mortality. One‐month and one‐year mortality rates were lowest in patients without anemia and highest in those with severe anemia. Our data demonstrate that early identification and optimization of preoperative anemia may reduce postoperative complications and potentially improve survival outcomes in patients undergoing surgery for spinal metastases.

## Abstract (highlights)

What's new?

High perioperative morbidity in patients undergoing surgery for spinal metastases highlights the need to identify modifiable risk factors, particularly the underexplored role of anemia. This study assessed associations between perioperative hemoglobin levels and complications, recurrence, and mortality in patients treated surgically for spinal metastases. Risk of hospital‐acquired complications was elevated in association with preoperative anemia, with mortality highest in severely anemic patients. By contrast, higher hemoglobin levels were protective. No associations were detected with surgical complications. The results highlight the importance of early detection and management of preoperative anemia to enhance postoperative recovery and survival in patients with spinal metastases.

## Graphical abstract

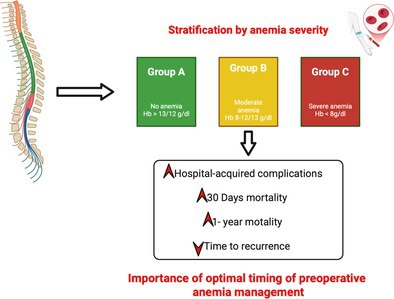

**Abbreviations**
AE: Adverse events
ASA: American Society of Anesthesiology
ASIA: American Spinal Injury Association
BMI: body mass index
CCI: Charlson comorbidity index
CI: confidence interval
HAC: hospital‐acquired complication
ICU: intensive care unit
IMC: intermediate care station
KPS: Karnofsky Performance Scale
OR: odds ratio
OS: overall survival
PE: pulmonary embolism
PSI: patient safety indicator
SD: standard deviation
SM: spinal metastasis

## 1 Introduction

Systemic cancer remains one of the leading causes of mortality worldwide, and more than one third of patients who die from malignancies are found on autopsy to have metastases to the spinal column [1, 2, 3]. Advances in modern, targeted oncologic therapies have extended patient survival but have simultaneously led to an increased incidence of spinal metastases [2, 4, 5, 6]. Their management requires a clearly defined and well‐coordinated multidisciplinary therapy algorithm, in which surgical resection plays an established central role in preserving quality of life—primarily by maintaining mobility, reducing pain, and enabling optimal administration of adjuvant chemotherapy and radiotherapy [7, 8].

Surgical options for managing spinal metastases encompass a spectrum, from transpedicular biopsy combined with vertebroplasty/kyphoplasty [9, 10], to neurosurgical decompression of the spinal canal [11], and/or combined with minimally invasive percutaneous surgical procedures [12], open dorsal instrumentation with augmented screws [13, 14], at times necessitating anterior‐lateral spine stabilization [15, 16], Despite the therapeutic benefits [17, 18], resection of spinal metastases remains a technically demanding and high‐risk procedure, frequently associated with extensive soft‐tissue dissection, prolonged operative times, and substantial intraoperative blood loss [19]. On the one hand, resection improves overall survival for many patients [11, 20], On the other hand, patients with spinal metastases often show poor general condition and suffer from comorbidities. Consequently, timely identification of potentially modifiable risk factors is essential, as their appropriate correction may substantially reduce the likelihood of adverse outcomes [21, 22].

In this context, perioperative anemia has increasingly come into focus as a potentially modifiable risk factor. Anemia continues to represent a significant clinical challenge due to its multifactorial etiology, often insufficient therapeutic management, and its well‐documented association with poorer outcomes and reduced survival [23]. Nevertheless, data specifically evaluating its prognostic relevance in patients undergoing surgical treatment for spinal metastases remain limited.

Therefore, the present study was designed to assess the impact of preoperative anemia and postoperative changes in hemoglobin levels following resection of spinal metastases on postoperative outcomes and patient mortality. By identifying the relationship between perioperative hemoglobin dynamics and surgical outcomes, we aim to contribute to strategies that improve preoperative optimization and facilitate timely postoperative intervention in patients undergoing surgery for spinal metastases.

## 2 Methods

### 2.1 Patients and Inclusion Criteria

This single center cohort study is based on consecutive patients aged > 18 years. We included all patients who underwent primary neurosurgical treatment for spinal metastases between 2013 and 2025 at our University Hospital Bonn center.

In total, 279 patients were identified and included in this study. Clinical data collected included age, sex, preoperative and postoperative hemoglobin (grams per deciliter), body mass index (BMI), Charleson comorbidity index (CCI) [24, 25, 26], Karnofsky Performance Scale (KPS) (categorizing patients into KPS ≥ 70% or KPS < 70%, as previously described [27, 28, 29, 30]), number and localization of tumor infiltrated segments, primary tumor type, history of chemotherapy or radiation therapy before surgery, American Society of Anesthesiology (ASA) physical status score. Additionally, functional status measured using the American Spinal Injury Association (ASIA) score [31] was recorded. Surgery‐related data included neurosurgical treatment, duration of operation and estimated intraoperative blood loss. Early postoperative complications were assessed using a publicly available list of adverse events introduced by the Agency for Healthcare Research and Quality and the Center for Medicare and Medicaid Services, and referred to as patient safety indicators (PSIs) and hospital‐acquired complications (HACs) [32, 33, 34, 35]. PSIs were classified as spinal surgery‐related complications. As described elsewhere, perioperative complications were defined as any postoperative adverse events, with or without further surgical intervention, occurring within 30 days of the initial surgery [36]. Overall survival was calculated from the date of surgical spinal metastases resection until death, as previously described [21].

### 2.2 Exclusion Criteria

We excluded patients without a complete clinical and neurological medical history as well as patients who had less than 1‐year follow‐up. Furthermore, patients were excluded who were not classified as operable.

### 2.3 Patients Groups

Patients were retrospectively classified into three groups according to their preoperative and postoperative hemoglobin level, using the World Health Organization's definition of anemia [37].

Group A consisted of patients without anemia (Hb > 13/12 g/dL), group B contained patients with mild to moderate anemia (Hb 8–12/13 g/dL), and group C contained patients with severe anemia (Hb < 8 g/dL).

Patient classification into anemia severity groups was based on the hemoglobin level measured at initial presentation before any transfusion. Hemoglobin values obtained after preoperative transfusion were not considered for group allocation. Postoperative hemoglobin values were recorded 3–4 days after surgery to minimize the potential dilutional effect of perioperative fluid administration on the assessment of postoperative anemia. Patients with preoperative erythrocytosis were not included in the study cohort.

### 2.4 Statistical Analysis

All data analysis was performed using IBM SPSS Statistics (version 25, IBM Corp., Armonk, NY). In the cases of categorical variables, data are given as percentages and numbers. Quantitative, normally distributed data are presented as mean values ± standard deviation (SD), while non‐parametric data are summarized by median values [first quartile–third quartile]. After normality testing via the Shapiro–Wilk test, continuous normally distributed data were compared using *t*‐tests, while the Kruskal‐Wallis test was used for non‐parametric data. Nominal data were tested between groups using Fisher's exact test and in the case of multinomial data with a chi‐squared test. A *p* value < 0.05 was considered statistically significant (two sided). We also conducted an univariate and a multivariate analysis in order to identify independent pre‐ and perioperative predictors. The baseline characteristics of the cohort were summarized using descriptive statistics.

Using logistic regression analysis, first binary and then multinominal, we investigated independent risk factors associated with perioperative anemia and postoperative complications, as well as mortality in the first month and the first year after resection of spinal metastases; odds ratios (ORs) with 95% confidence intervals (CIs) were calculated. The graphs were created using GraphPad Prism Version 10.6.1.

## 3 Results

### 3.1 Patient Characteristics and Demographic Data

A total of 279 patients fulfilled the inclusion requirements and were therefore selected for further statistical analysis. The median age was 66 years (interquartile range 58–75 years). Overall, 62.4% of patients were male and 37.6% female. The median BMI was 25 (interquartile range 23–27). At the time of surgery, 70.3% of patients had already been diagnosed with disseminated systemic disease. Lung cancer was the most common primary site (23.3%) of tumor, followed by other types (20.8%) and prostate cancer (19.4%). Preoperatively, 52% of patients had undergone systemic therapy and 18.6% of patients had received radiotherapy. More detailed baseline patient characteristics are shown in Table 1.

**TABLE 1:** Baseline characteristics of study population, *n* = 279.

Variable	*n* (%) or median [IQR]
Age, years	66 [17]
Sex	
Male	174 (62.4)
Female	105 (37.6)
Mean hemoglobin concentration (Group A)	13.8 g/dL [1.70]
Mean hemoglobin concentration (Group B)	10.9 g/dL [1.85]
Mean hemoglobin concentration (Group C)	7.6 g/dL [0.75]
BMI	25 [4]
ASA	
ASA ≤ 2	107 (38.4)
ASA > 2	172 (61.6)
Previous chemotherapy	146 (52.3)
Previous radiotherapy	52 (18.6)
Primary malignant tumor	
Lung	65 (23.3)
Prostate	54 (19.4)
Kidney	23 (8.2)
Breast	29 (10.4)
Gastrointestinal tract	29 (10.4)
Plasmacytoma	21 (7.5)
Others	58 (20.8)
Preoperative ASIA classification	
ASIA (D, E)	198 (71)
ASIA (A, B, C)	81 (29)
Segments infiltrated	
≤ 2 segments	172 (61.6)
≥ 3 segments	107 (37.9)
Karnofsky Performance Scale	
KPS ≥ 70%	187 (67)
KPS ≤ 70%	92 (33)
Disseminated systemic disease	196 (70.3)
Surgical methods	
Decompression	107 (38.4)
Decompression and stabilization	172 (61.6)
Use of anticoagulants	66 (23.7)
Postoperative transfusion	73 (26.2)
Operative time, minutes	184 [126]
Estimated blood loss, ml	600 [700]
Length of stay, days	12 [11]

*Note:* Categorical variables are shown as number (%) and continuous variables as median (IQR).

Abbreviations: ASA, American Society of Anesthesiology; ASIA, American Spinal Injury Association; BMI, body mass index; IQR, interquartile range; KPS, Karnofsky Performance Scale.

### 3.2 Disease and Patient‐Related Characteristics Dependent on Perioperative Anemia

The mean preoperative hemoglobin levels across all groups was 11.92 ± 2. The mean hemoglobin concentration was 13.8 g/dL (IQR 12.95–14.65) in group A, when stratified by sex, hemoglobin values were 13.9 g/dL (IQR 1.7) in males and 13.4 g/dL (IQR 1.55) in females, 10.9 g/dL (IQR 9.98–11.83) in pooled group B, and 7.6 g/dL (IQR 7.23–7.98) in pooled group C.

Preoperative anemia was absent in 41.9% of the 279 patients, mild to moderate anemia was present in 53.4% of patients, and severe anemia was present in 4.7% of patients.

The distribution of anemia varied across tumor types, with the highest proportion of mild to moderate anemia observed in patients with lung and prostate cancer and the highest proportion of severe anemia in patients with gastrointestinal cancer and prostate cancer.

To identify factors associated with the presence of preoperative anemia, a univariate analysis was performed, using the Kruskal–Wallis test for continuous variables and the *χ* ^2^ test for categorical variables. ASA score (*p* < 0.001), BMI (*p* = 0.005), and the presence of disseminated metastatic disease (*p* = 0.033) were identified as significant predictors of preoperative anemia in the overall analysis. In contrast, patient age, sex, and the use of preoperative therapy were not significantly associated with the presence of preoperative anemia in this univariate analysis. Following the identification of statistically significant differences in the overall analysis, post hoc analyses were subsequently performed to further explore and delineate the specific between‐group differences.

Preoperative hemoglobin levels were significantly associated with the occurrence of HACs (Pearson *χ* ^2^(2) = 13.712, *p* = 0.001). Lower hemoglobin levels were associated with a higher incidence of HAC.

### 3.3 Multivariate Analysis Identifies Preoperative Anemia as an Independent Predictor of Mortality and Complications

In the subsequent binomial logistic regression model, preoperative anemia remained a significant predictor of HACs (*p* = 0.002).

Preoperative anemia was also significantly associated with functional outcomes as represented by the KPS (*p* < 0.001), as well as neurological outcomes, represented by the ASIA score (*p* = 0.013). However, the association between preoperative hemoglobin levels and patient safety outcomes was not statistically significant (*p* = 0.236). Additionally, no statistically significant association was found between preoperative hemoglobin and surgical‐site complications (*p* = 0.400). (Table 2).

**TABLE 2:** Multivariable binomial logistic regression model assessing the association between preoperative hemoglobin and postoperative outcome.

Variable	β	SE	Wald χ2	df	OR	95% Cl	*p* (sig.)
HAC	0.838	0.276	9.241	1	2.31	1.35–3.97	0.002
PSI	−0.294	0.248	1.402	1	0.745	0.458–1.213	0.236
SSC	−0.310	0.369	0.707	1	0.733	0.356–1.511	0.400
KPI	1.083	0.299	6.235	1	2.954	1.643–5.311	< 0.001
ASIA	0.824	0.330	6.235	1	2.279	1.194–4.352	0.013

Abbreviations: ASIA, American Spinal Injury Association; Cl, confidence interval; df, degrees of freedom; HAC, hospital‐acquired complications; KPS, Karnofsky Performance Scale; OR, odds ratio; PSI, patient safety indicator; SE, Standard error; sig. significance; SSC, surgical site complication; 𝛽, 𝛽 Coefficient.

**FIGURE 1:**
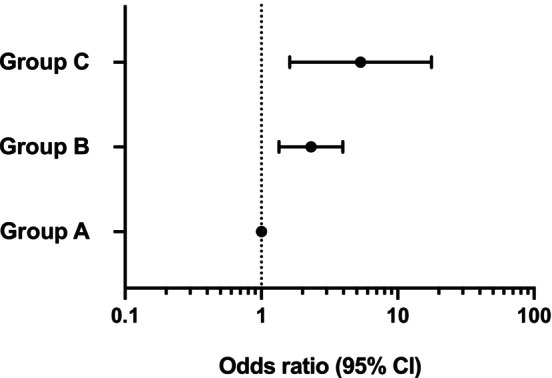
Forest plot of odds ratios (OR) with 95% confidence intervals for preoperative hemoglobin categories as predictors of hospital‐acquired complications. Compared with the reference category (Group A), mild to moderate anemia was associated with higher odds of hospital‐acquired complications (OR = 2.31; 95% CI: 1.35–3.97; *p* = 0.002), while severe anemia showed an even stronger association (OR = 5.33; 95% CI: 1.61–17.66; *p* = 0.006). Group A: Patients without anemia; Group B: Patients with mild to moderate anemia; Group C: Patients with severe anemia.

Only tumor involvement of more than two segments showed an independent association with surgical‐site complications (*p* = 0.040). Further statistical analysis compared outcomes, represented by HACs, across all three categories of preoperative anemia. Compared to the reference group (group A), patients with moderate anemia (group B) had a significantly increased risk of complications (*p* = 0.002). Patients with severe anemia (group C) were at an even higher risk (*p* = 0.006). (Figure 1).

In the multivariate logistic regression, in addition to preoperative anemia being a significant independent predictor of postoperative HACs, significant predictors were age over 65 years (*p* = 0.050) and receipt of preoperative chemotherapy (*p* = 0.011). Interestingly, perioperative transfusion did not emerge as a predictor of postoperative complications in our analysis. (Table 3).

**TABLE 3:** Multivariable logistic regression analysis of predictors of hospital‐acquired complications.

Predictor	OR	95% Cl	*p*
Mild to moderate anemia	2.146	1.158–3.977	0.015
Severe anemia	7.006	1.705–28.779	0.007
Age > 65	1.773	0.999–3.146	0.050
Sex	1.192	0.666–2.131	0.554
ASA score	1.542	0.835–2.848	0.167
Operation duration	1.000	0.997–1.004	0.852
Preoperative chemotherapy	2.299	1.206–4.384	0.011
Preoperative anticoagulation	0.465	0.246–0.878	0.018
Perioperative transfusion	1.003	0.518–1.942	0.994

Abbreviations: ASA, American Society of Anesthesiology; CI, confidence interval; OR, odds ratio.

A minimally significant statistical association of postoperative hemoglobin was observed with both HAC (*χ* ^2^ = 6.007, degrees of freedom [df] = 2, *p* = 0.050) and ASIA scores (*χ* ^2^ = 5.951, df = 2, *p* = 0.051), whereas no significant association was observed with spinal surgery‐related complications (*χ* ^2^ = 0.899, df = 2, *p* = 0.638) or with KPS values (*χ* ^2^ = 1.139, df = 2, *p* = 0.566). Given the observed borderline statistical significance of these associations and the fact that postoperative hemoglobin was analyzed across multiple categories, additional post hoc analyses were performed to identify the specific groups contributing to the overall effect. Post hoc contrasts revealed that patients in group C (with the lowest postoperative hemoglobin) had a significantly higher likelihood of poor outcome compared with the reference group (*p* = 0.020). Group B's results were not significantly different from the reference group's (*p* = 0.238). Furthermore, a multivariate analysis was conducted to assess the independent contribution of postoperative hemoglobin to the outcome, while simultaneously controlling for relevant covariates. Postoperative anemia, categorized into three groups with Group A (Patients without Anemia) as reference, was not independently associated with postoperative complications in multivariable logistic regression (overall Wald test; *p* = 0.190). Compared with non‐anemic patients, both anemia groups showed a trend toward higher odds of complications (Group B: OR 3.28; *p* = 0.072; Group C: OR 3.62; *p* = 0.097), although these differences did not reach statistical significance.

With the aim of evaluating a dynamic indicator of perioperative blood loss and physiological stress, which may have greater prognostic value than isolated absolute hemoglobin levels, we analyzed the impact of hemoglobin change on the occurrence of postoperative complications and mortality. Hemoglobin change was defined as postoperative hemoglobin minus preoperative hemoglobin. Logistic regression analysis showed that hemoglobin change did not have a statistically significant effect on the likelihood of postoperative complications (*p* = 0.389, OR = 1.008, 95% CI 0.990–1.026).

The 30 days mortality rates were 4.3% in group A, 14.1% in group B, and 23.1% in group C. A statistically significant difference was observed among the groups (*p* = 0.011; and p: 0.008–0.013). To evaluate the association between preoperative hemoglobin levels and early postoperative mortality, a logistic regression analysis was performed. The overall model showed statistically significant results (*p* = 0.018). Compared with group A patients without anemia, those in group B had a 3.7‐fold increased risk of 30 days mortality (OR = 3.675, 95% CI 1.342–10.067, *p* = 0.011), while the risk was even higher in Group C, with a 6.7‐fold increase (OR = 6.720, 95% CI 1.397–32.325, *p* = 0.017).

Cox proportional hazards analysis demonstrated that preoperative hemoglobin levels significantly affected 1 month survival (*p* = 0.040). Patients with mild to moderate anemia (group B) had a significantly higher hazard of mortality within the first postoperative month (HR = 3.83, *p* = 0.014) than group A patients. Patients with severe anemia (group C) showed an elevated hazard ratio as well (HR = 5.01), although this did not reach statistical significance (*p* = 0.063).

**FIGURE 2:**
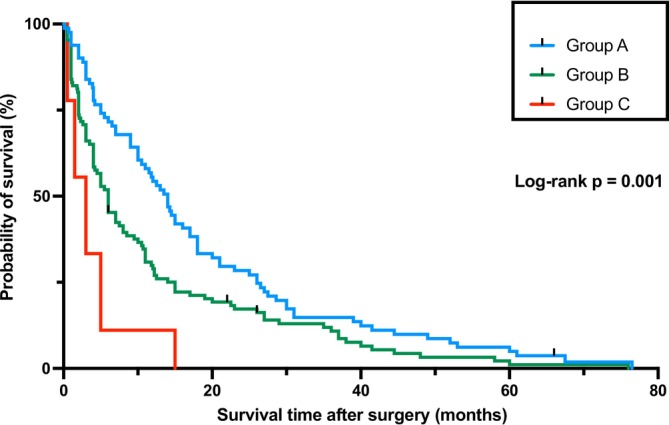
Kaplan–Meier estimates of overall survival (OS) according to preoperative hemoglobin levels. OS was significantly longer in patients without anemia (Group A) compared with patients with mild‐to‐moderate anemia (Group B) and severe anemia (Group C). Patients with severe anemia (Group C) had the shortest OS. Survival curves differed significantly among groups (log‐rank *p* = 0.001). The number of patients at risk is shown below the survival curves. Group A: Patients without anemia; Group B: Patients with mild‐to‐moderate anemia; Group C: Patients with severe anemia.

With regard to the impact of preoperative hemoglobin levels on overall mortality, Kaplan–Meier analysis demonstrated significant differences in overall survival according to preoperative hemoglobin levels. Patients without anemia had a median overall survival of 14 months, whereas those with moderate and severe anemia showed markedly shorter survival of 5.5 and 3 months, respectively (Figure 2). These differences were statistically significant (log‐rank *χ* ^2^ = 19.453, *p* < 0.001; Breslow *χ* ^2^ = 23.456, *p* < 0.001).

Furthermore Cox proportional hazards analysis showed that preoperative hemoglobin levels were significantly associated with overall mortality (*p* < 0.001). Compared to patients with normal hemoglobin, moderate anemia was associated with a 1.6‐fold increased hazard of death (HR = 1.59, *p* = 0.002), and severe anemia with a 3.6‐fold increased hazard (HR = 3.57, *p* < 0.001).

As the most important independent predictors of poorer overall survival, in addition to preoperative anemia, multivariable analysis identified a higher ASA score, lower BMI, and perioperative transfusion.

Postoperative hemoglobin level was not a statistically significant predictor of mortality in either the first month or the first year after tumor resection. After adjusting for covariates in the multinomial regression analysis, neither postoperative hemoglobin (OR = 0.308, 95% CI 0.04–2.41, *p* = 0.262; OR = 0.453, 95% CI 0.04–5.56, *p* = 0.536) nor the dynamic change in hemoglobin (OR = 0.971, 95% CI 0.945–0.998, *p* = 0.031) emerged as independent risk factors for 30 days mortality and overall mortality.

### 3.4 Recurrence‐Free Survival

When we analyzed frequency of local recurrence according to preoperative hemoglobin levels, the proportion of patients with recurrence was similar across all groups: 51 patients (43.6%) in group A, 64 patients (43.0%) in group B, and five patients (38.5%) in group C. Chi‐square analysis showed no significant association between preoperative hemoglobin levels and local recurrence (*χ* ^2^ = 0.126, df = 2, *p* = 0.939).

**FIGURE 3:**
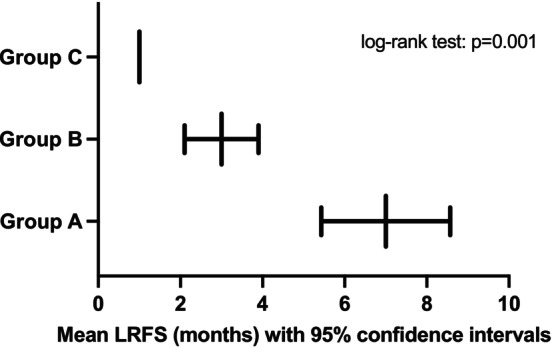
Mean local recurrence‐free survival (LRFS) by anemia severity, presented as mean values with 95% confidence intervals. LRFS: Local recurrence‐free survival; Group A: Patients without anemia; Group B: Patients with mild to moderate anemia; Group C: Patients with severe anemia.

On the other hand, the analysis of local recurrence‐free survival (LRFS) revealed notable differences among the groups. Patients without anemia had the longest mean LRFS, averaging 7 months (95% CI 5.43–8.57). In patients with mild to moderate anemia, the mean LRFS was 3 months (95% CI 2.10–3.90), while the shortest LRFS of 1 months (95% Cl not estimate) was observed in the group with severe anemia. According to the log‐rank (Mantel–Cox) test, these differences were statistically significant (*χ* ^2^ = 13.03, *p* = 0.001). (Figure 3).

We performed a complete sensitivity analysis excluding all patients with multiple myeloma and plasmacytoma (*n* = 258) and repeated the identical statistical workflow. The main findings remained unchanged. Preoperative hemoglobin categories remained significantly associated with HACs, functional status (Karnofsky Performance Index), neurological status (ASIA score), mortality (monthly and 1‐year), and time to local recurrence, while no significant association was observed with the Patient Safety Index (PSI) or surgical‐specific complications. In multivariable analysis, preoperative hemoglobin remained independently associated with HAC, with increased odds in Group B (OR 2.310, 95% CI 1.270–4.201) and Group C (OR 5.235, 95% CI 1.348–20.337), compared with patients without anemia. A tabulated overview of the statistical analyses for the revised sensitivity cohort is provided in the Supporting Information (Tables S1–S3).

## 4 Discussion

The timely identification of perioperative risk factors associated with the treatment remains a key prerequisite within the therapeutic concept for managing spinal metastases. By reducing the incidence of complications and optimizing therapeutic procedures, the likelihood of a more favorable outcome increases, particularly in oncology patients whose life expectancy is already inherently limited [6]. Our clinical research at our university spine center focuses on clinical parameters that have a significant impact on survival in patients with surgically treated spinal metastases [21, 22, 38]. This retrospective single center cohort study aimed to analyze the prognostic value of perioperative anemia as a surrogate factor for predicted complications in patients with surgically treated spinal metastases.

Numerous studies have suggested that low hemoglobin levels may be associated with an increased risk of postoperative complications, which has led to the recognition of hemoglobin as an independent prognostic marker for surgical outcomes [39, 40]. Therefore, monitoring and timely correction of perioperative hemoglobin levels are not merely technical aspects of perioperative care, but essential components of a comprehensive strategy aimed at reducing risk and improving both functional and oncological outcomes [41].

Preoperative hemoglobin reflects the patient's baseline physiological status and compensatory reserves. Patients with lower preoperative hemoglobin enter surgery with a reduced oxygen‐carrying capacity, which increases the risk of perioperative hypoxia, further contributing to pre‐existing hypoxic–ischemic damage to vital organs. In addition, the surgical resection itself promotes chronic inflammation and hypercoagulation, collectively increasing the risk of postoperative complications [42].

We identified preoperative hemoglobin concentration as a significant independent predictor of HACs. A clear risk gradient was observed depending on the severity of anemia. Patients with mild to moderate anemia had a more than 2‐fold increased risk of developing postoperative complications acquired during hospitalization, whereas patients with severe anemia had a more than 5‐fold higher risk compared with individuals without anemia.

In contrast to our findings, a retrospective study by Wang et al. reported that postoperative hemoglobin was linearly associated with the risk of composite adverse events [43]. We believe that this discrepancy may stem from the substantial variability of postoperative hemoglobin measurements, which are influenced by intraoperative blood loss, transfusions, and hemodilution. Consequently, these values may represent a poor indicator of a patient's true physiological reserve.

To better elucidate individual hemoglobin dynamics, we evaluated its perioperative changes as a potential predictive marker. However, contrary to our initial assumptions, we found that hemoglobin change was not a statistically significant predictor of postoperative complications. These results differ from those reported by Spolverato et al., who identified a significant association between ΔHb ≥ 50% and a nadir hemoglobin level < 7 g/dL with postoperative complications in a large cohort of more than 4500 patients. The discrepancy between the two studies may be attributed to differences in study design, including variations in patient cohort, types of surgical procedures, and overall perioperative risk profiles [44]. In spinal tumor surgery, wound complications—primarily surgical‐site infections—are among the most frequent postoperative issues, with the highest reported incidence reaching 11.2% [45]. Our findings indicate that perioperative anemia was not a significant predictor of surgical‐site complications. Instead, the extent of disease involvement and the scope of the surgical procedure emerged as more important determinants of postoperative surgical complications.

Our study also demonstrated that a higher degree of preoperative anemia has a significant impact on postoperative functional outcomes, as reflected by KPS and ASIA scores. It is assumed that the underlying mechanism of this phenomenon is tissue hypoxia, which disrupts cellular metabolism, limits regeneration, and consequently leads to poorer neurological and functional recovery [46]. Although spinal metastases are common, the available data on predictors of short‐term and long‐term prognosis in these patients remain limited [47]. To complement existing knowledge, numerous prognostic scoring systems have been developed with the aim of improving the accuracy of outcome prediction and understanding the patient's prognosis [48]. Available data indicate that the average overall survival of patients with metastatic spinal lesions is approximately 10 months [49].

The results of our study demonstrated that the risk of 1 month and 1 year mortality is inversely proportional to the severity of anemia. The results are consistent with previously published studies indicating that higher preoperative hemoglobin levels in patients undergoing surgery correlate with improved overall survival compared to patients with hemoglobin values below 6 g/dL. However, Carson et al.'s study focused on patients with cardiovascular diseases, and the hemoglobin threshold they used was considerably lower than the one applied in our study [50]. With regard to survival time as an outcome measure, preoperative anemia has consistently been identified as a factor associated with unfavorable clinical outcomes. Its effect must be clearly distinguished from that of perioperative transfusion, given that transfusion itself is associated with increased mortality and a higher risk of secondary complications [51, 52]. In our study, we found that preoperative anemia represented an independent risk factor, whereas perioperative transfusion did not demonstrate a statistically significant influence on the incidence of complications or on mortality. As is well known, reduced hemoglobin concentration limits oxygen transport, impairs peripheral tissue oxygenation, and leads to secondary tissue hypoxia [53]. Hypoxia, on the other hand, may serve as an important stimulus that initiates cellular remodeling processes and thus contributes to patient recovery [46]. However, the question arises as to whether the same mechanisms apply to oncology patients. In healthy tissue, adequate oxygenation is maintained at hemoglobin levels above 8 g/dL. Although oxygenation of normal tissue decreases between 8 g/dL and 4 g/dL, it generally remains sufficient due to physiological compensatory mechanisms, primarily increased blood flow. In contrast, locally advanced tumors are unable to compensate for the reduced oxygen supply and therefore cannot prevent the development of hypoxia. Experimental data show that oxygenation of tumor tissue is markedly compromised and that hypoxia further intensifies at hemoglobin levels below 10–12 g/dL [54]. An increasing number of studies indicates that hypoxia within tumor tissue may directly contribute to resistance to radiotherapy or chemotherapy by limiting the availability of oxygen, which is essential for the cytotoxic effects of these treatment modalities [55]. Additionally, hypoxic conditions may indirectly enhance radioresistance and chemoresistance by inducing proteomic and genomic alterations that accelerate malignant progression, reduce local disease control, and promote metastasis [56, 57].

To the best of our knowledge, no prior research has systematically evaluated the impact of varying degrees of anemia on the incidence of local recurrence, LRFS, overall clinical outcomes, or overall survival in patients who have undergone resection of spinal metastases.

## 5 Conclusion

Preoperative hemoglobin levels proved to be an important and independent predictor of postoperative complications, functional recovery, and overall survival in our cohort of patients undergoing surgery for spinal metastases. Mild to moderate preoperative anemia was associated with a significantly higher risk of unfavorable outcomes, and this was particularly the case with severe preoperative anemia. On the other hand, the absence of anemia had a protective effect.

In contrast, postoperative hemoglobin values and perioperative hemoglobin changes did not show a significant association with adverse outcomes or mortality. Additionally, preoperative anemia was linked to a tendency for earlier local recurrence, further confirming its prognostic value in oncologic patients and suggesting that it may influence the pace of disease progression.

## 6 Limitations

It should be noted that this study has several limitations. First, as a retrospective investigation, it is subject to selection bias and the influence of factors that were not fully controlled. Second, the number of patients with severe anemia in our cohort was small, which may considerably affect the interpretation and generalizability of the results. Third, patients with multiple myeloma were included in the analysis; however, multiple myeloma is, by definition, a systemic hematologic disorder rather than a metastatic disease, and typically demonstrates a more favorable prognosis and better therapeutic response compared to other malignancies. To address this limitation, we performed a sensitivity analysis excluding patients with multiple myeloma and plasmacytoma (*n* = 21).

As the findings did not differ from those observed in the initial cohort, these patients were retained in the final analysis.

Additionally, neuro‐oncologically relevant parameters and factors such as perioperative chemotherapy and/or radiation are important clinical aspects that can positively or negatively influence the postoperative course as well as complications; these aspects were not recorded in our data and should be taken into account in future clinical prospective studies.

## Author Contributions

**Marija Janjic:** conceptualization, methodology, software, validation, formal analysis, data curation, writing – original draft, writing – review and editing. **Logman Khalafov:** conceptualization, software, validation, data curation, writing – review and editing. **Juliane Dittmer:** writing – review and editing. **Saif‐Eldin Abedellatif:** writing – review and editing. **Tim Lampmann:** writing – review and editing. **Harun Asoglu:** writing – review and editing. **Mohammed Jaber:** writing – review and editing. **Haitham Alenezi:** writing – review and editing. **Muriel Heimann:** writing – review and editing. **Matthias Schneider:** writing – review and editing. **Motaz Hamed:** writing – review and editing. **Marcus Thudium:** writing – review and editing. **Hartmut Vatter:** investigation, writing – review and editing. **Mohammed Banat:** conceptualization, methodology, software, data curation, supervision, resources, project administration, validation, investigation, funding acquisition, writing – review and editing, visualization, writing – original draft.

## Ethics Statement

The study adhered to the ethical principles outlined in the 1964 Helsinki Declaration and received approval from the Ethics Committee of the University Hospital Bonn (reference no. 067/21). Given the retrospective nature of the study, the acquisition of informed consent from participants was not necessary.

## Conflicts of Interest

The authors declare no conflicts of interest.

## Supporting information


**Table S1:** Baseline characteristics of the study population, *n* = 258.
**Table S2:** Multivariable binomial logistic regression model assessing the association.
**Table S3:** Multivariable logistic regression analysis of predictors of hospital‐acquired.

## Data Availability

The datasets generated and/or analyzed during the current study are available from the corresponding author on reasonable request.
