# Supplementary material for: Prognostic Value of Perioperative Anemia in Neurosurgical‐Oncological Treatment of Spinal Metastases
Source: Int J Cancer. 2026 Apr 27;159(6):1391–400. doi: 10.1002/ijc.70513 (PMC13397302; doi:10.1002/ijc.70513)
Supplement: Supplementary file 1 — Table S1: Baseline characteristics of the study population, n = 258. Table S2: Multivariable binomial logistic regression model assessing the association. Table S3: Multivariable logistic regression analysis of predictors of hospital‐acquired. [file IJC-159-1391-s001.pdf]

Prognostic value of perioperative anemia in neurosurgical-oncological treatment of spinal metastases

Marija Janjic, L. Khalafov, J. Dittmer, S. Abedellatif, T. Lampmann, H. Asoglu, M. Jaber, H. Alenezi, M. Heimann, M. Schneider, M. Hamed, M. Thudium, H. Vatter, M. Banat

Table of Contents

Table S1. Baseline characteristics of the study population, n=258

Table S2. Multivariable binomial logistic regression model assessing the association between preoperative hemoglobin and postoperative outcome

Table S3. Multivariable logistic regression analysis of predictors of hospital-acquired complications

Table S1. Baseline characteristics of study population, n=258

Categorical variables are shown as number (%) and continuous variables as median (IQR).

Variable	n (%) or median [IQR]
Age, years	66 [16]
Sex	
Male	161 (62.4)
Female	97 (37.6)
Mean hemoglobin concentration (Group A)	13.8 g/dL [1.70]
Mean hemoglobin concentration (Group B)	10.9 g/dL [1.77]
Mean hemoglobin concentration (Group C)	7.7 g/dL [0.80]
BMI	25 [4]
ASA	
ASA < = 2	97 (37)
ASA > 2	161 (62.4)
Previous chemotherapy	134 (51.9)
Previous radiotherapy	48 (18.6)
Primary malignant tumor	
Lung	64 (24.8)
Prostate	55 (21.3)
Kidney	23 (8.9)
Breast	29 (11.2)
Gastrointestinal tract	28 (10.9)
Others	59 (22.9)
Preoperative ASIA classification	

ASIA (D, E)	194 (75.2)
ASIA (A, B, C)	64 (24.8)
Segments infiltrated	
≤ 2 segments	158 (61.2)
≥ 3 segments	100 (38.8)
Karnofsky Performance Scale	
KPS ≥ 70%	170 (65.9)
KPS ≤ 70%	88 (34.1)
Disseminated systemic disease	188 (72.9)
Surgical methods	
Decompression	100 (38.8)
Decompression and stabilization	158 (61.2)
Use of anticoagulants	62 (24)
Postoperative transfusion	64 (24.8)
Operative time, minutes	183.50 [123.75]
Estimated blood loss	600 [700]
Length of stay, days	12 [11]

ASIA, American Spinal Injury Association; ASA, American Society of Anesthesiology; BMI, Body Mass Index; IQR, interquartile range; KPS, Karnofsky Performance Scale.

Table S2. Multivariable binomial logistic regression model assessing the association between preoperative hemoglobin and postoperative outcome

Variable	β	SE	Wald χ^2	df	OR	95% CI	P (sig.)
HAC	0.837	0.305	7.531	1	2.310	1.270–4.201	0.006
PSI	-0.216	0.273	0.624	1	0.806	0.471-1.377	0.429
SSC	-0.574	0.372	2.389	1	1.776	0.857-3.680	0.122
KPI	0.763	0.308	6.128	1	2.145	1.172-3.926	0.013
ASIA	0.682	0.030	4.263	1	1.978	1.035-3.778	0.039

Table S3. Multivariable logistic regression analysis of predictors of hospital-acquired complications

Predictor	OR	95% CI	p value
Mild to moderate anemia	2.310	1.270-4.201	0.006
Severe anemia	5.235	1.348-20.337	0.017
Age > 65	1.812	1.005-3.267	0.048
Sex	1.275	0.712-2.281	0.413
ASA score	1.420	0.774-2.604	0.257
Operation duration	1.001	0.998-1.005	0.340
Preoperative chemotherapy	2.304	1.211-4.385	0.011
Preoperative anticoagulation	0.489	0.255-0.936	0.031
Perioperative transfusion	0.892	0.462-1.721	0.733

ASA: American Society of Anesthesiology; CI: confidence interval; OR: odds ratio;
